# Measuring the WHO Global Breast Cancer Initiative Pillars’ key performance indicators in Sub-Saharan Africa: experience in the African Breast Cancer—Disparities in Outcomes hospital-based cohort study

**DOI:** 10.1016/j.eclinm.2025.103104

**Published:** 2025-02-13

**Authors:** Pauline Boucheron, Annelle Zietsman, Angelica Anele, Awa U. Offiah, Moses Galukande, Groesbeck Parham, Leeya Pinder, Tingting Mo, Milena Foerster, Joachim Schüz, Benjamin O. Anderson, Mary Nyangasi, Isabel dos-Santos-Silva, Valerie McCormack

**Affiliations:** a International Agency for Research on Cancer (IARC/WHO), Environment and Lifestyle Epidemiology Branch, Lyon, France; b AB May Cancer Centre, Windhoek Central Hospital, Windhoek, Namibia; c Federal Medical Centre, Owerri, Nigeria; d Abia State University Teaching Hospital, Aba, Nigeria; e College of Health Sciences, Makerere University, Kampala, Uganda; f Department of Obstetrics and Gynaecology, School of Medicine, University of North Carolina, Chapel Hill, NC, USA; g University of Washington, Seattle, WA, USA; h City Cancer Challenge (C/Can), Geneva, Switzerland; i World Health Organization, Geneva, Switzerland; j Department of Non-Communicable Disease Epidemiology, London School of Hygiene and Tropical Medicine (LSHTM), London, United Kingdom

**Keywords:** Global Breast Cancer Initiative, Key performance indicators, Breast cancer, Sub-Saharan Africa

## Abstract

**Background:**

The World Health Organization Global Breast Cancer Initiative aims to reduce breast cancer (BC) mortality through three pillars, whose key performance indicators (KPIs) and benchmarks are: (KPI-1) ≥60% BC diagnosed at early stage (I/II), (KPI-2) all suspected BC diagnosed ≤60 days from health system presentation, and (KPI-3) ≥80% of BC patients completing recommended treatment. We aimed to inform measurement of these KPIs in the context of a multi-country hospital-based study.

**Methods:**

We included all women who participated to the African Breast Cancer—Disparities in Outcomes (ABC-DO) prospective cohort study (excluding South Africa), recruited between 2014 and 2017, across five population-race groups spanning low to high survival: Nigeria, Uganda, Zambia, Namibian black and Namibian non-black women. Follow-up was up to five years post-diagnosis. For each KPI, we reported challenges, assumptions and consistencies in measuring them; completeness and group-level estimations of each KPI were assessed using descriptive analyses. To evaluate their discriminatory ability, we assessed group-level correlations between KPI estimates and five-year net survival.

**Findings:**

KPI-1 was extracted from study or medical records for 1389/1473 (94%). KPI-2 relied upon the woman's recall of her date of first contact with the healthcare system and a pathology date, both of which were available for 1222/1473 (83%) but inconsistent for 114/1222 (9.3%). KPI-3, estimated using dates of receipt of multiple therapies from medical records and patient interviews over 12 months, was estimated for 1129/1188 (95%), but uncertain in 113/1129 (10%). For each population group, KPIs achievements were similar for KPI-1 and KPI-2, at 22–49%, and lowest for KPI-3 (<30%). Highest KPIs values were observed in Namibian non-black women who had the highest survival.

**Interpretation:**

Data collection systems specifically set up for prospective hospital-based studies can be used to collect the necessary data to measure these three GBCI KPIs.

**Funding:**

10.13039/100000054National Cancer Institute (United States).

**Research in context**

**Evidence before this study**

With population growth and ageing, the breast cancer (BC) burden is increasing globally, both in terms of incidence and mortality, especially in low- and middle-income countries. The World Health Organization's Global Breast Cancer Initiative (GBCI) aims to reduce population-level age-standardized BC mortality rates by 2.5% per year through 2040, via three pillars of action, namely (i) health promotion for early detection, (ii) timely diagnosis, and (iii) comprehensive BC management, each of which is measured by a Key Performance Indicator (KPI) with a minimum benchmark required to reduce BC mortality. We searched PubMed with the following search strategy: ((“KPI” OR “Key Performance Indicator∗”) AND (“Global Breast Cancer Initiative” OR “GBCI”) AND measur∗ AND method∗) without restrictions on publication dates or language. Up to January 2, 2025, only one study—which aimed to produce national estimates across 21 Asian countries—had reported on the feasibility of measuring these KPIs. This study used secondary data of literature published prior to the publication of the GBCI implementation framework, thus, definition of the KPIs varied according to setting and data source. Other studies reporting on methods to measure the KPIs are needed, notably in Sub-Saharan Africa (SSA) given the large survival gains needed in this region.

**Added value of this study**

This study is the first to present the methods, information requirements and considerations to measure each of the GBCI pillars KPIs in SSA by using data from the African Breast Cancer—Disparities in Outcomes prospective cohort study, which was conducted in tertiary hospitals located in countries with different healthcare systems. It also identifies the third KPI as the one with the lowest completion, thus needing a greater investment to ensure it is captured. We also investigated the associations of population-level KPIs estimates with five-year survival.

**Implications of all the available evidence**

The findings demonstrate that it is feasible to establish appropriate data collection systems in tertiary hospitals to measure the GBCI pillars KPIs in a standardised manner across SSA through targeted patient interviews coupled with standardised recording of key clinical parameters in medical records. Collection of KPI data will need to rely on multiple data sources, especially for pillars 2 and 3. The positive correlations between population-level KPIs estimates and 5-year survival point towards their relevance to inform BC control. Systematic measurement, monitoring and dissemination of the KPIs to policymakers and other stakeholders will be key to support decision making aimed at improving local BC control.

## Introduction

Breast cancer (BC) has been increasingly recognized as a cancer control priority. BC is currently both the most commonly diagnosed and leading or second cause of cancer deaths in females, with 2.3 million new cases and 666,000 deaths worldwide in 2022.^1^ Following the World Health Organization (WHO) 2017 Cancer Prevention and Control Resolution calling for greater investments into cancer, the WHO launched the Global Breast Cancer Initiative (GBCI) in 2021 to reduce population-level age-standardized BC mortality rates by 2.5% per year through 2040, by promoting multisectoral partnerships, sustainable capacity building and developing monitoring systems for decision making.^2^^,^^3^

In 2023 the GBCI published an implementation framework formed of three pillars of action across the BC care continuum, each measured by a benchmarked evidence-based key performance indicator (KPI): (pillar 1) “health promotion for early detection”, i.e., primary prevention and early diagnosis (pre-diagnostic interval) (KPI-1: ≥60% of invasive BC stage I/II at diagnosis); (pillar 2) “timely breast diagnosis”, i.e., diagnostic interval (KPI-2: diagnostic work-up—diagnostic evaluation, imaging, tissue sampling and pathology—within 60 days of first presentation to the healthcare system for all women); and (pillar 3) “comprehensive BC management”, i.e., treatment interval (KPI-3: ≥80% of BC patients undergo their recommended treatment without abandonment).^4^, ^5^, ^6^ Through these pillars, progress to reduce advanced stage at diagnosis and improve treatment quality should ultimately lower population-level BC mortality rates.

Measuring and monitoring a country's progress towards these “60-60-80” KPIs benchmarks is essential to guide governmental policymakers to prioritize BC control actions. A recent study reported upon the possibilities and limitations of estimating GBCI pillars KPIs across 21 Asian countries, with estimates ideally sourced from national level data; however, availability of KPI data from national or regional cancer registries was generally low (e.g., KPI-1 data was missing for 14 countries; and data for KPI-2 and KPI-3 were missing for nearly all countries), as previously reported in studies embedded within cancer registries in Sub-Saharan Africa (SSA).^7^, ^8^, ^9^ In SSA, although imperfect, hospitals may represent a valuable and feasible setting to measure the KPIs.

The data-rich multi-country African Breast Cancer—Disparities in Outcomes (ABC-DO) hospital-based BC cohort study has previously reported on multiple dimensions of BC survival and its determinants and those articles should be utilized for the corresponding BC control research.^10^, ^11^, ^12^, ^13^, ^14^ In contrast, the present paper focuses on exemplifying how the three main GBCI pillars KPIs might be measured in a hospital research setting; (i) we document the methods and considerations in defining and measuring the KPIs; (ii) visually display an informative summary of the KPIs estimates to facilitate communication of progress and identify gaps in BC control; and (iii) assess the discriminatory ability of the KPIs between populations known to have differing survival. The results endeavour to strengthen future data collection to measure the KPIs.

## Methods

### Study design and participants

ABC-DO was set up in major hospitals across five SSA countries of different cultures, human development index and health systems. These were: (Nigeria) Federal Medical Center, Owerri and the Abia State University Teaching Hospital and private Maranatha clinic, Aba; (Namibia) AB May Cancer Center of the Windhoek Central Hospital; (Uganda) the Mulago Hospital complex including the Uganda Cancer Institute, Kampala; (Zambia) University Teaching Hospital, Lusaka, and Kabwe General Hospital, Kabwe, and (South Africa (SA)) the Chris Hani Baragwanath Hospital. Per protocol, all women—defined as per sex assigned at birth—who presented at one of these hospitals with suspicious, clinically, or histologically confirmed incident BC were invited to participate, regardless of ethnicity, residential location, or ability to pay for treatment.^15^ Sociodemographic, BC and treatment characteristics of the subset of the ABC-DO participants included in this KPI assessment are presented in Appendix 1. The present analysis was restricted to the four ABC-DO countries which used the same data collection system and baseline interview, thus excluding SA (n = 689 women). Women with suspected prevalent BC (i.e., histology or self-reported diagnosis ≥2 years before presenting to the study hospital, n = 63) were also excluded, leaving 1473 women included. These 1473 women were recruited from 8th September 2014 to 18th August 2017. Their maximum potential 5-year follow-up was to 18th August 2022.

### Ethics

The ABC-DO study was approved by the International Agency for Research on Cancer ethics committees and all the local ethics committees, as previously published.^16^ All women provided their oral and written (or thumbprint) informed consent.

### Data sources and data collection system in ABC-DO

The same standardized real-time data collection system utilizing mHealth technology was used across the four included countries. In each country, a team of research assistants was provided with smartphones, and trained in mobile data collection. Extensive data on the whole BC care continuum was obtained via (i) retrospective recall by women from symptom(s) self-recognition to diagnosis, and then (ii) prospectively collected through treatment and survival via three data sources: (a) one baseline face-to-face interview at first post-diagnosis visit to the study hospital including collection of women's and next-of-kin's (NOK) contact details for follow-up, (b) tri-monthly follow-up phone calls to the woman or her NOK if she could not be reached, and (c) study proforma inserted into medical records from the study onset.^17^ Answering questions was made mandatory (with a “I prefer not to answer” option) and implemented checks immediately identified data collection errors, which minimized both missing data and data management needs. From first recruitment to end 2021, this data collection system was managed on a tailormade platform programmed by Mobenzi.^18^ Each interviewer automatically received an updated list of follow-up calls due. Women reported dead were automatically removed from future follow-up lists. After the platform closed, follow-up lists were maintained centrally on a similar system made on KoboCollect.^19^

### Statistics

All analyses, performed on STATA v17, were stratified by population groups (i.e., country and, within Namibia, race). Table 1 details calculation methods for the three KPIs. To visually display each KPI progress towards their benchmark, we used radial semi-pie charts, divided into three equal slices—one per KPI—with radial distances proportional to progress towards 100%, a fully coloured slice area indicating 100%. For aim (iv), survival analyses were performed on the time since diagnosis scale with follow-up starting at baseline and ending at the earliest date of (i) death, (ii) last live contact, or (iii) administrative censoring (5-year post-diagnosis). Population-level 5-year net survival (NS) which accounted for year-age-country-specific background mortality rates was also plotted against each KPI estimate.^20^ Unadjusted Cox models with baseline hazards stratified by population group (i.e., country, and within Namibia, self-reported race) were fitted to examine associations between individual-level KPI measurements and all-cause mortality as cause of death was not available. Cox proportional hazards assumption was checked graphically using Schoenfeld residuals. Unadjusted logistic regression models were used to examine relationships between achievement of what we considered to be a binary interpretation of the KPIs at the individual level, i.e. having stage I/II BC, being diagnosed ≤60 days and completing treatment. Note that at the individual level, the KPI benchmarks for pillars 1 and 3 no longer appear. Note also that the follow-up time included periods beyond 1st June 2020 (i.e., when the Covid-19 pandemic's first mortality wave hit Africa) for 384 (26%) participants; however, sensitivity analyses censoring on 1st June 2020 hardly altered 5-year survival estimates (not shown). Furthermore, none of the KPI data quality or completeness was affected by Covid.

**Table 1:** Measurement methods for GBCI pillars key performance indicators, in ABC-DO.

GBCI pillar KPI	WHO GBCI definition and benchmark	ABC-DO application	Essential variables	Data source(s)	Considerations	Decisions	Calculation methods	Exclusions	Sensitivity analysis
KPI-1 (pillar 1)	Proportion of invasive BC cases diagnosed at stage I or II, excluding unstaged and stage 0, benchmark of 60%	Proportion of women with stage I/II BC at baseline (i.e., prior to treatment initiation)	BC stage (based on TNM coded according to UICC/AJCC or essential staging systems)	Medical records (study proforma)	Diagnosis is often clinical (i.e., 17% of women were solely clinically diagnosed in ABC-DO)	None	No. women with stage I/II divided by no. women with invasive BC	- Missing stage - Stage 0	None
KPI-2 (pillar 2)	Diagnosis within 60 days (two months) of initial presentation to the healthcare system, benchmark of 100%	Proportion of women histologically diagnosed within 60 days of first presentation to the healthcare system (i.e., time to specimen sampling + duration of diagnostic work-up)	- Date of first visit to the healthcare system - Date of histological diagnosis	- Baseline interview when the patient recalled her date of first health care contact - Pathological report	- Differences in the definition of “healthcare system” (formal sector only or consideration of informal sector (i.e., traditional healers) as part of the system) - Recalled data (i.e., date of first visit) may be subject to measurement error	- Utilized date of first visit to formal healthcare system for Namibia and Zambia, and to either formal or informal sector for Nigeria and Uganda (based on MoH reports) - Date of histological diagnosis: latest available date of (i) pathology report, (ii) laboratory receipt, or (iii) specimen sampling	No. women with a diagnostic interval ≤60 days divided by no. women with non-missing length of diagnostic interval	- Missing date of histology - Inconsistencies: Reported date of first presentation after that of diagnosis (main analysis)	Inclusion of women with a recalled date of first presentation after that of diagnosis, and consider their date of first visit as the earliest available date of receipt of diagnosis (i) specimen sampling, (ii) laboratory receipt, or (iii) pathology report (i.e., sole consideration of duration of diagnostic work-up)
KPI-3 (pillar 3)	Proportion of BC patients who complete the recommended therapy without abandonment, benchmark of 80%	Proportion of women with non-metastatic disease who fully completed their study-determined guideline-recommended treatment	All indicators needed to inform treatment regimen as per guidelines. Currently: - Age - HR status or tumour subtype - T, N, M - Grade - Types of treatment received (SX, CH, ET, RT, IT) with dates of initiation and last receipt - CH regimen, no. cycles - Date of metastatic diagnosis - Vital status	- Baseline and trimonthly follow-up interviews - Medical records (study proformas)	- Requires many variables to be collected - Ascertainment of metastatic status can be difficult - IHC needed to ascertain HR status not always available in settings - RT and IT generally not available - Ascertainment of completion of ET requires long-follow-up. Also, adherence to ET is difficult to ascertain. - Unavailability of cause of early treatment cessation: difficult to differentiate treatment abandonment from cessation for medical reason.	- ET considered indicated in women with unknown or HR positive BC - RT—where available, and ET were considered as binary variables (received yes/no) - RT and IT only considered in settings where it was generally available - Treatment completion was considered up to the date of death or of metastases development	No. of women with non-metastatic disease who fully completed their recommended treatment divided by the no. of women with non-metastatic disease	- Missing stage - Unknown treatment status	Exclusion of women who died within six months of baseline, as treatment received may have been with palliative intent

ABC-DO, African Breast Cancer—Disparities in Outcomes; AJCC, American Joint Committee on Cancer; UICC, Union for International Cancer Control; BC, breast cancer; CH, chemotherapy; ET, endocrine therapy; GBCI, Global Breast Cancer Initiative; HR, hormonal receptor; IHC, immunohistochemistry; IT, immunotherapy; KPI, Key Performance Indicator; MoH, Ministry of Health; N/A, not applicable; RT, radiotherapy; SX, surgery.

### Role of the funding source

The funder had no role in the design and conduct of the study; collection, management, analysis, and interpretation of the data; preparation, review, or approval of the manuscript; and decision to submit the manuscript for publication. Authors were not precluded from accessing data in the study, and they accept responsibility to submit for publication.

## Results

**Fig. 1:**
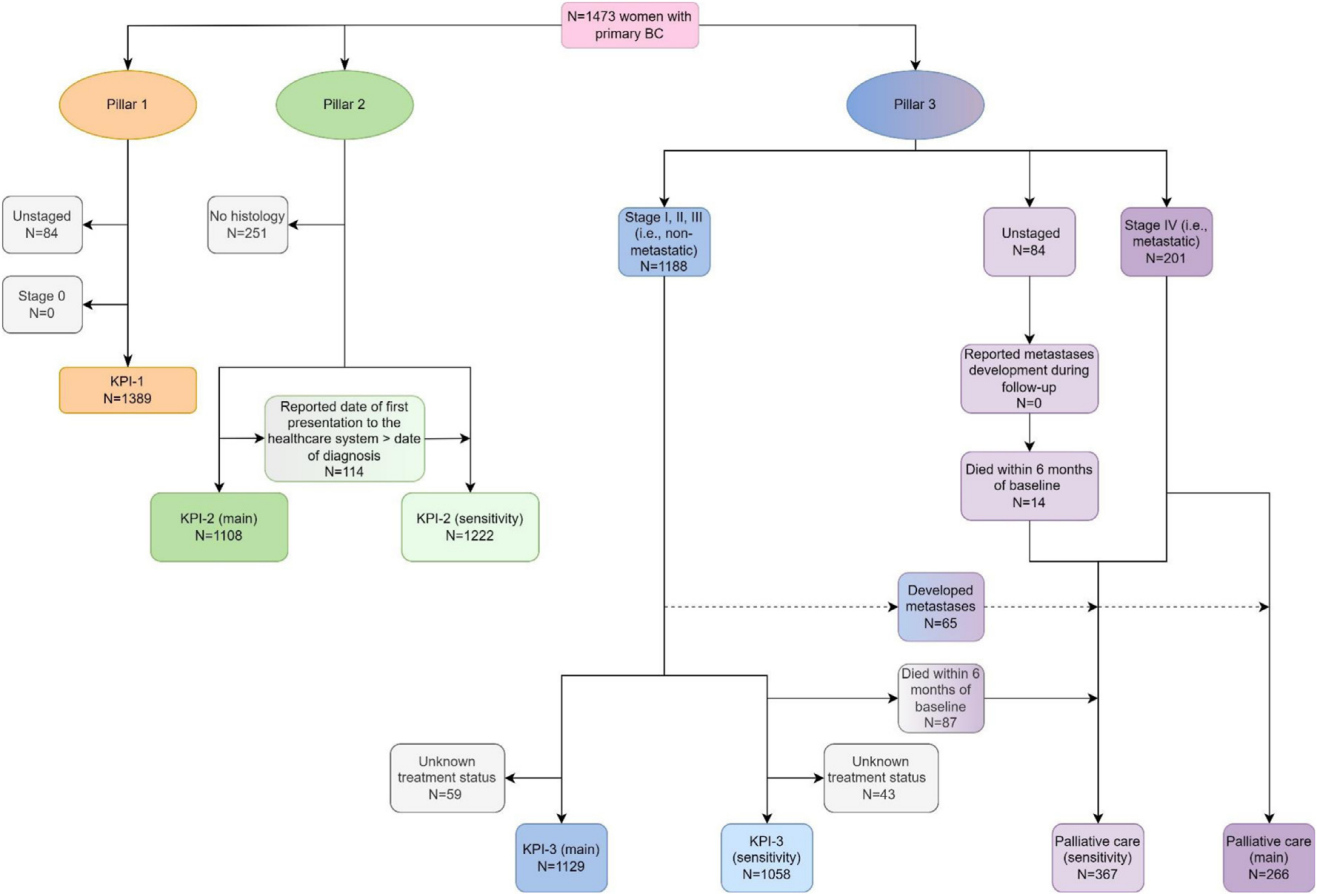
**Flowchart for participant inclusion for various calculations of each GBCI key performance indicator**. BC, breast cancer; GBCI, Global breast cancer initiative; KPI, Key Performance Indicator; Sens, sensitivity.

Fig. 1 provides a flowchart of women included for each KPI measurement.

### Pillar 1 KPI

#### Definition

The GBCI defines KPI-1 as the “proportion of invasive BC cases diagnosed at stages I or II, excluding unstaged and stage 0” (60% benchmark).^4^ We defined KPI-1 as the proportion of women with early-stage BC assessed at baseline (i.e., prior to treatment initiation) among those with non-missing stage (Table 1).

#### Methods

TNM staging—the only variable needed to inform KPI-1—was retrieved from medical records or the study stage proforma and coded according to the 7th version of the American Joint Committee on Cancer (AJCC) BC staging system using anatomic staging.^21^

#### Estimates

**Fig. 2:**
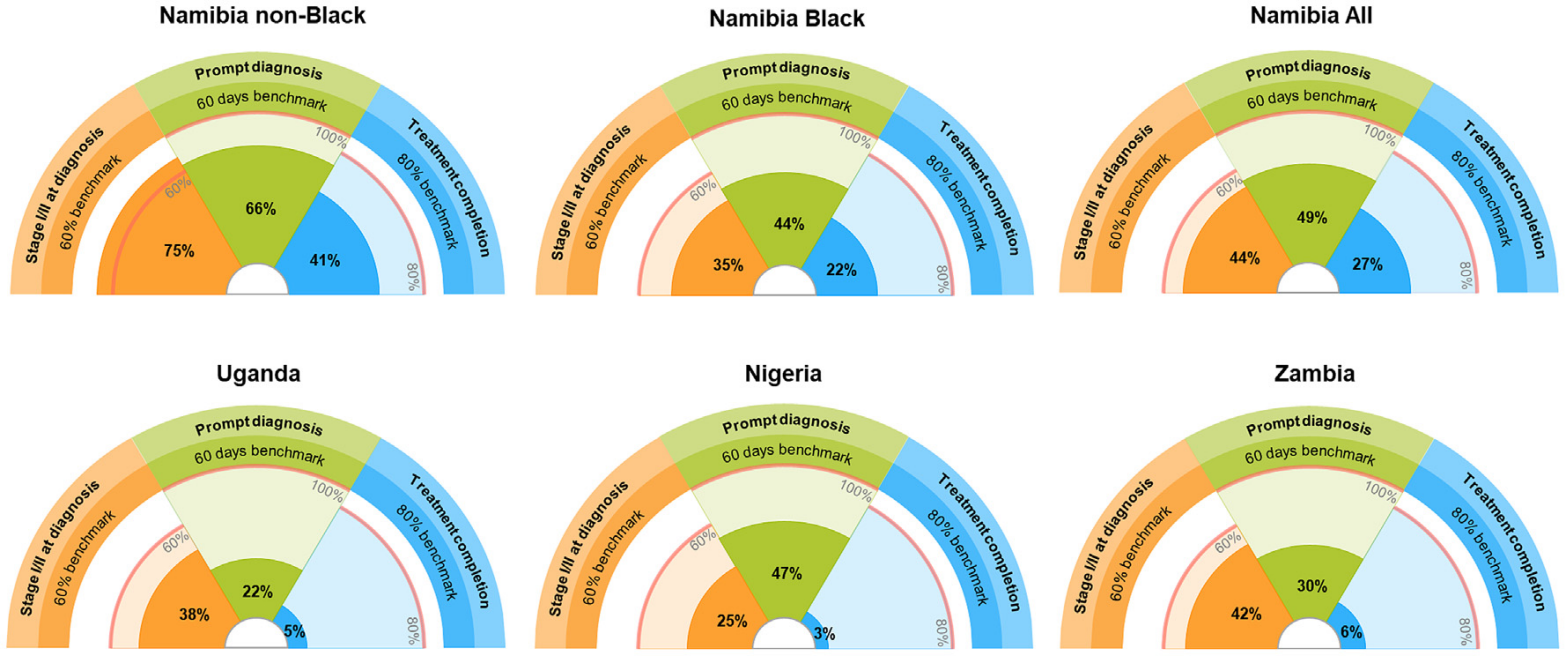
**Visualization of the progress towards the GBCI pillars' KPIs benchmarks by country and race, in ABC-DO**. ABC-DO, African Breast Cancer—Disparities in Outcomes; GBCI, Global breast cancer initiative; KPI, Key Performance Indicator. N.B: Clockwise, the arcs represent pillars 1, 2 and 3, with the fully shaded area representing progress towards the benchmark indicated by the red line.

Stage data were available for 1389/1473 (94%) women (min: 175/199 (88%), Zambia; max: 477/477 (100%), Namibia), and when reported (n = 1112/1389, 80%), 87% relied upon clinical or imaging methods (mostly ultrasound) (Fig. 1, Table 2, Appendix 2). None of the Black African population groups achieved the 60% stage I/II benchmark (KPI-1 range: from 88/353 (25%), Nigeria, to 74/175 (42%), Zambia); however, it was surpassed in non-Black Namibians (75%) (Fig. 2).

**Table 2:** GBCI pillar key performance indicators estimates, by country and race, in ABC-DO (main analysis).

ABC-DO, African Breast Cancer—Disparities in Outcomes; GBCI, Global breast cancer initiative; KPI, Key Performance Indicator.

### Pillar 2 KPI

#### Definition

Defined by the GBCI as a “diagnosis within 60 days of initial presentation to a healthcare system” irrespective of diagnostic outcome (100% benchmark), we defined KPI-2 as the proportion of women with malignant BC receiving a histological diagnosis within 60 days of first contact with the healthcare system regardless of the visit outcome (Table 1).^4^

#### Methods

Assessment of KPI-2 requires, in chronological order, the dates of (i) first contact with the healthcare system, and (ii) confirmatory diagnosis. The former was retrospectively recalled at baseline when women were asked about visits to “nurses, doctors, traditional healers, clinics, hospitals or any health provider at all, before coming to this hospital”, its location and type—precision may vary with durations from first contact with the health system to the baseline interview (≥6 months: 15%; ≥12 months: 20%). The definition of “healthcare system” may vary between settings, and was defined according to each country's Ministry of Health, including both the formal and informal sectors in Nigeria and Uganda, and the formal sector only in Namibia and Zambia.^22^, ^23^, ^24^, ^25^ We chose to base the second date (confirmatory BC diagnosis) solely on histological confirmation (n = 1222/1473 women, 83%), which was retrieved from pathological records and defined as the latest available date of (i) biopsy (n = 5, 0.45%), (ii) laboratory receipt (n = 18, 1.6%), or (iii) pathology report (n = 1075, 97%)—10 (0.90%) women had an unspecified date of histological diagnosis (Appendix 2). Overall, 114/1222 (9.3%) women reported inconsistent dates—date of first presentation after that of pathological confirmation [>30 days: 52/1222 (4.3%), mostly in Nigeria: 32/149 (21%)]. Thus, KPI-2 was analysed (i) excluding these 114 women and, as sensitivity analysis, (ii) including them considering their date of first presentation as the earliest available date of receipt of diagnosis—their diagnostic period therefore solely reflected duration of the biopsy work-up—time prior to biopsy was omitted (Table 1).

#### Estimates

Amongst 1108/1222 (91%) women histologically diagnosed, KPI-2 ranged from 22% (n = 76/342) in Uganda to 49% (n = 224/455) in Namibia (Black vs non-Black Namibians: 44% vs 66%) (Fig. 2, Table 2). In sensitivity analysis (n = 1222 women), while KPI-2 estimates changed marginally in most countries, in Nigeria where most patients resided near the hospital, KPI-2 increased by 12% (59%, n_sens_ = 120/205 vs 47%, n_main_ = 70/149) (Appendices 3 and 4).

### Pillar 3 KPI

#### Definition

The GBCI implementation framework defines KPI-3 as the “proportion of BC patients who receive their recommended treatment to completion without abandonment” (80% benchmark), where “completion” is the “fulfilment of all components of the therapeutic sequence” and “abandonment” signifies “failure to complete a treatment regimen due to reasons other than medical indications for treatment disruption”.^4^ We defined KPI-3 as the proportion of women with non-metastatic disease who fully completed guideline-recommended treatment (Table 1).

#### Methods

KPI-3 is complex to measure considering the numerous variables needed to inform treatment indication as per the concurrent NCCN Harmonized guidelines for SSA—used in ABC-DO—or any other region, and the extended treatment period. Required data include stage, tumour grade, metastases development, hormonal receptor (HR) and vital statuses, and treatment (types, dates, and, for chemotherapy, number of cycles and regimen) (Table 1).^26^ In ABC-DO, these were extracted from (i) medical records (study proformas for stage and treatment details), (ii) follow-up interviews, and (iii) baseline interviews for women who had undergone treatment before presenting at the study hospital—detailed characteristics of treatment received by ABC-DO women, and their compliance with NCCN Harmonized guidelines for SSA have been previously described.^27^ Access to immunohistochemistry (i.e., ascertainment of HR status) to inform indication of endocrine therapy (ET), and to radiotherapy and immunotherapy were very limited in Nigeria, Uganda and Zambia; also, long-term adherence to ET could not be ascertained. Thus, we considered (i) these three treatment types as binary variables (received yes/no) with dates of initiation, (ii) ET as indicated in BC with unknown or positive HR status, and (iii) radiotherapy and immunotherapy in Namibia only. Also, reasons of early treatment cessation to distinguish abandonment from other causes were not systematically available in ABC-DO. Treatment completion was considered up to metastatic diagnosis, defined as the date of (i) diagnosis (cf. Pillar 2)—for women with stage IV disease at presentation, or (ii) metastases discovery reported in medical records—if metastases developed during follow-up. Palliative care was considered indicated from metastatic diagnosis; however, palliative care data were scarce in ABC-DO possibly due to a lack of reporting or access and were not considered for KPI-3 calculation. KPI-3 was analysed in women with non-missing treatment status diagnosed with non-metastatic BC. A sensitivity analysis was performed excluding those who died within six months of baseline as they may have received care with palliative intent. As only 3/84 unstaged women had non-missing treatment information and their metastatic status was unknown, these were not included in KPI-3 analysis.

#### Estimates

Of 1129/1188 (95%) women with non-metastatic disease and known treatment status, 992 (88%) initiated BC treatment (Appendix 2). Of all KPIs, KPI-3 estimates were the lowest in all countries (min: 9/288 (3.1%), Nigeria; max: 109/400 (27%), Namibia (Black: 23% vs non-Black: 41%)); however, treatment completion was uncertain for 113 (10%) women (range: Namibia: 3/400 (0.8%); Uganda: 80/294 (27%)). Of 1102/1129 (98%) women who reported a first contact with the healthcare system before receiving treatment, 39/288 (14%) (Uganda) to 192/397 (48%) (Namibia, Black: 42% vs non-Black: 66%) initiated treatment timely (i.e., ≤90 days of first presentation) (Table 2, Fig. 2). Amongst 266 women with metastatic disease, 123 (46%) had some palliative care information reported, and 120 (45%) received any palliative care (min: 13/70 (19%) Uganda; 85/117 (73%) Namibia) (Appendix 2). Results remained similar in sensitivity analysis (Appendices 3 and 4).

### Impact of KPIs on 5-year survival

**Fig. 3:**
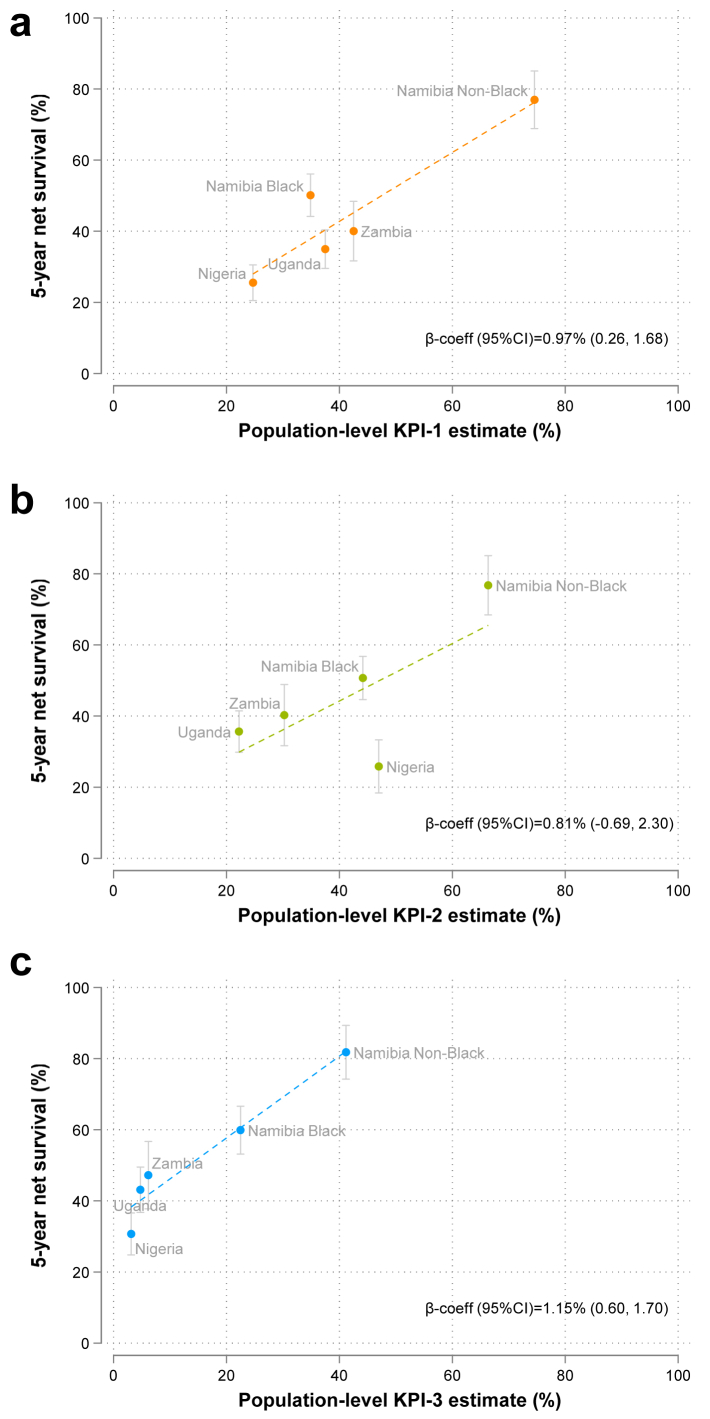
**Scatter plots of five-year net survival vs KPI progress by GBCI pillar KPI, in ABC-DO**. ABC-DO, African Breast Cancer—Disparities in Outcomes; CI, confidence interval; KPI, Key Performance Indicator. Scatter plots showing five-year net survival against country- and race-specific a) KPI-1, b) KPI-2, and c) KPI-3 estimates. N.B: For each KPI, analyses were performed amongst the population group subset where both the KPI and net survival could be measured. N.B2: Beta-coefficients (95% CI) obtained from unadjusted linear regression models, representing the slope of the fitted linear regression line, and interpreted as the absolute percentage point (p.p.) increase in 5-year net survival, per 1 absolute p.p. increase in KPI estimate.

**Fig. 4:**
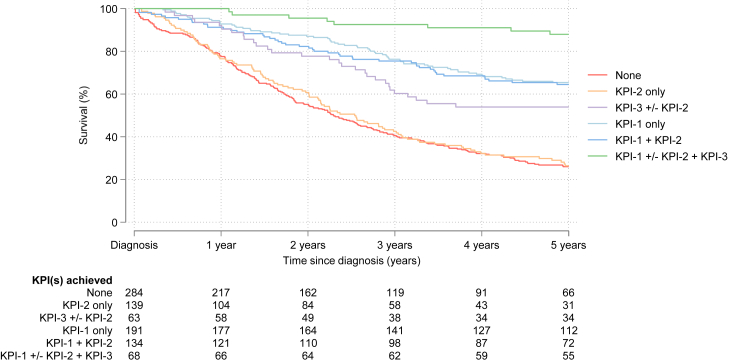
**Overall survival to five years by the individual-level co-achievement of early stage, diagnosis ≤60 days and complete treatment, i.e. the closest binary individual-level dichotomization of each GBCI KPI(s), in non-metastatic women in ABC-DO∗**. ABC-DO, African Breast Cancer—Disparities in Outcomes; KPI, Key Performance Indicator. ∗This analysis was restricted to women for whom all three KPIs could be calculated (i.e., 879 women affected with non-metastatic disease). N.B: No KPI achieved (hazard ratio (95% CI): 1 (Ref)), KPI-2 only (0.98 (0.76, 1.25), KPI-3 ± KPI-2 (0.62 (0.41, 0.93)), KPI-1 only (0.34 (0.26, 0.46)), KPI-1 ± KPI-2 (0.38 (0.27, 0.53)), and KPI-1 ± KPI-2 + KPI-3 (0.15 (0.07, 0.31)). Unadjusted Hazard ratios (95% CI) obtained from Cox models stratified on population group (country and, within Namibia, race).

KPI-1 and KPI-3 were selected by the GBCI as they are well-established strong determinants of survival in BC patients, whereas KPI-2 is a strong determinant of KPI-1 (unadjusted odds ratio (95%CI) in ABC-DO: 1.50 (1.16, 1.93), p = 0.002, not in tables). When we assessed whether achievement of the KPI benchmarks was also associated with survival in country-race groups with greatly differing levels, while all KPIs correlated positively with 5-year net survival (Fig. 3a, b and c), the steepest gains in NS were seen for KPI-1 and KPI-3 changes (Fig. 3a and b). Deviating from GBCI, if we drop the benchmark and instead consider the individual-level co-achievement of early stage, diagnosis ≤60 days and complete treatment (among 879 non-metastatic women), it is clear that survival is better in individual women who achieve these (Fig. 4 & Appendix 5). All-cause mortality rates were almost three and two-fold lower in those who achieved early stage (targeted within KPI-1) or completed treatment (included in KPI-3), respectively, than in those who did not (hazard ratio (95% CI): 0.32 (0.27, 0.38)) and 0.65 (0.47, 0.89), data not shown), and were lowest when both early stage and complete were fulfilled (0.15 (0.07, 0.31)).

## Discussion

This study is the first to evaluate both the practicability of measuring the three GBCI KPIs in tertiary hospitals, and their correlation with survival both at the individual and population level, within a well-funded multi-country research context in SSA. The ABC-DO's data collection system which was set up for a hospital-based prospective cohort study was suitable for measuring the three GBCI KPIs in a standardized manner across four SSA settings with different healthcare systems, as indicated by the high percentage of complete data, and the expected variability in KPIs estimates between countries and races known to differ in survival after a BC diagnosis.

KPI-1's definition and measurement are reasonably straightforward. Two considerations are needed. First, whether the unstaged patients represent a random sample of all patients, which we assumed by omitting unstaged women in KPI-1 calculations; however, diagnostic procedures to prove the presence of metastatic disease may not be performed in low- and middle-income countries (LMICs) due to out-of-pocket expenses that may be viewed as unnecessary and/or unhelpful. Thus, some unstaged women may be frail very late-stage patients and KPI-1 values might be overestimated, especially when the proportion of missing stage is high. Second, BC might be under-staged where staging relies mostly on clinical exam or basic imaging (ultrasound). The TNM staging system and version used should be cited to enhance between-settings comparability of KPI-1 estimates.

KPI-2—the diagnostic interval length—requires both precise definitions of and a chronological order in its start and end date. In the GBCI framework, the start date “first presentation to the healthcare system” is referred to both (i) the start of the diagnostic work-up and (ii) the first visit to a healthcare provider (HCP) after symptom(s) discovery.^4^ Whilst the latter description is relatively clear, when the diagnostic work-up commences is less so and might involve HCP at different levels of the healthcare system including community health workers performing a basic clinical breast exam. Considering the rationale for KPI-2 benchmark of 60 days, using the date of first visit to a HCP as the starting point of the diagnostic period seems most relevant.^28^ This start date could be captured by routinely asking women when they first presented to the healthcare system; while subject to random measurement error, reliability of this recalled information was acceptable in ABC-DO—starting from this date, a longer diagnostic interval was one of the strongest determinants of late-stage diagnosis.^12^^,^^13^ Also, definition of the first contact with the system may require some local adaptations—we included visits to the informal sector in countries where it is officially part of the healthcare system. The end date of this interval is the date of diagnosis. A range of definitions could be applied here too. In the perfect situation, this would be the date of pathological diagnosis including appropriate immuno-histochemistry. A more basic definition might also include diagnoses based upon clinical or imaging only. We attempted to apply a definition that was realistic to attain, thus providing a useable indicator that could be used to measure and promote progress. Complete health passports, and investment in e-health records allowing analysis of all health system contacts may enhance precision of KPI-2 assessment.

KPI-3 requires numerous time-updated data on the multi-modal therapies typical of BC received over many months or years; hence its measurement is complex. Where access to immunohistochemistry—essential to determine treatment indication—and of some treatment modalities are limited, KPI-3 assessment may need to start by measuring what is currently possible (i.e., completion of generally available BC treatment modalities in a setting) to obtain actionable information; also, treatment components could be evaluated separately. GBCI emphasises the need for multidisciplinary planned BC treatment based on resource-adapted guidelines, thus recommendations vary and need to be specified. Ideally, reasons for early treatment cessation (e.g., death, losses to follow-ups, medical decisions, lack of supplies) would be collected to distinguish abandonment from other causes. KPI-3 calculation may also need to be restricted to non-metastatic patients as properly capturing palliative treatment information can be challenging—frail metastatic patients may not reach the treatment hospital and may be visited at home or attend local hospitals.

Data collection systems set up for hospital-based studies can allow measurement of the three GBCI pillar KPIs, including in LMICs. Collection of KPIs data could be centralized in a country's main hospitals with the necessary BC diagnostic and treatment competencies, decentralized in facilities where at least one KPI can be measured (diagnostic centres for KPI-1 and KPI-2, treatment centres for KPI-3), or a mix of both approaches. While decentralization may enhance representativeness of KPIs estimates—which depends on the case mix and referrals patterns, it might require more follow-up and access to health records in hospitals beyond the initial diagnostic institution. Data collection would need to rely on multiple data sources, especially for pillar 2—recall data and pathological records were essential for measuring KPI-2—and pillar 3—BC treatment components might be provided by separate services. In each participating hospital, a team of professionals trained to collect and analyse the KPIs data could be responsible for regularly monitoring the KPIs on a sufficiently large random sample of BC patients. Freely available standardized real-time mHealth data collection forms embedding automatic data quality checks would reduce data management needs and considerably facilitate KPIs monitoring. Such a data collection system would require electronic devices with an internet connexion for data storage on a central secured server—the latter being generally available in secondary- or tertiary-level hospitals.

This study had several limitations. ABC-DO did not rely primarily on routine data collection by the participating hospitals but was set up as a separate research effort which involved specific data collection through targeted patient interviews, periodic standardised data abstraction from clinical notes and mHealth based active follow-up which ensured high data quality and very few dropouts (<10% at the end of the 5-follow-up year). A quarter of the participants had follow-up extending through the Covid-19 pandemic; however, this did not affect quality of collection of KPIs data, as women were all diagnosed (KPI-1 and KPI-2) three to six years prior to the start of the pandemic, and of those affected with non-metastatic BC (KPI-3) who initiated treatment, all had received surgery and finished chemotherapy; furthermore, in survival analysis, year-age-country-specific background mortality rates were applied. Lastly, KPIs estimates are not representative of the whole population of BC patients in each country, as these were collected in main hospital settings, and are likely overestimated, pertaining to the (i) non-inclusion of women who did not reach the study hospitals (e.g., frailty, lack of access, no referral, consultation in the private sector), and (ii) restriction of each KPI estimation to patients with non-missing information, both of which may have favoured the inclusion of women with better BC prognosis.

This study demonstrated the feasibility of measuring KPIs in LMICs, such as those in SSA, by setting up specific data collection systems in tertiary hospitals. The positive relationships between the KPIs estimates and 5-year survival point towards their relevance to inform BC control. An effort should be made to provide countries with readily implementable standardized indicators and data collection tools for measurement and reporting of KPIs requiring minimal maintenance and low resources to ensure their sustainability. Whenever KPIs are measured and reported it is essential to provide clear KPIs definitions with detailed calculations methods together with any decisions taken, and the evidence-generation behind them such as systematic or literature reviews. This will ensure transparency and will facilitate (i) optimization of KPIs measurement methods and (ii) interpretation of their estimates, ultimately improving their accuracy, reproducibility, and comparability both within- and between-settings. Standardized visualisation tools representing progress towards benchmark achievement (e.g., cf. Fig. 2) could be used to facilitate monitoring and dissemination of the KPIs estimates, and to inform key stakeholders on which interventions should be prioritised to improve local BC control. A separate study could evaluate palliative care in women affected with metastatic disease, as this requires specific data collection efforts. While this work reported on the three main and macro-level GBCI pillars KPIs, the GBCI is currently developing technical documents to guide its implementation; one recently published provides guidance for improving healthcare system navigation pathways to foster earlier detection, prompt diagnosis and better management of BC.^29^

In conclusion, in SSA, measuring GBCI KPIs in tertiary hospitals is possible, provided that appropriate adaptations to both existing data collection systems and KPIs definitions are made. Achieving higher KPIs values will ultimately translate into substantial reductions in BC mortality. Hence, monitoring KPIs, utilizing standardized ready-to-use sustainable data collection systems, is essential to inform local BC control plans of the progress being made towards that goal.

## Contributors

PB statistically analysed the data. PB, VM and IdSS had full access to the data, drafted the manuscript and were responsible to submit the manuscript. AA, AUO, AZ, MG, GP, LP, JS, VM, BOA and IdSS designed the study. TM contributed to the statistical analysis. All authors contributed to interpretation of the results and writing of the report, read and approved the final version of the manuscript. PB and VM verified all the data in the study.

## Data sharing statement

All data generated or analysed during this study are included in this published article and its supplementary information files. Further information is available from the study PIs (VM and IdSS) upon request to mccormackv@iarc.who.int.

## Declaration of interests

We declare no competing interests.
