# Supplementary material for: Measuring the WHO Global Breast Cancer Initiative Pillars’ key performance indicators in Sub-Saharan Africa: experience in the African Breast Cancer—Disparities in Outcomes hospital-based cohort study
Source: eClinicalMedicine. 2025 Feb 13;81:103104. doi: 10.1016/j.eclinm.2025.103104 (PMC11872636; doi:10.1016/j.eclinm.2025.103104)
Supplement: Supplementary material [file mmc1.docx]

**Table of Contents**

[Appendix 1. Characteristics of the population included in this study, by country and race 2](#_Toc185521633)

[Appendix 2. Supplementary data for the main analysis 3](#_Toc185521634)

[Appendix 3. Supplementary data for the sensitivity analyses 5](#_Toc185521635)

[Appendix 4. GBCI pillar key performance indicators estimates, by country and race, in ABC-DO (sensitivity analysis) 6](#_Toc185521636)

[Appendix 5. Patterns of co-KPI achievement, in ABC-DO 7](#_Toc185521637)

# **Appendix 1. Characteristics of the population included in this study, by country and race**

|  | | | **Namibia Black (n=367)** | **Namibia non-Black (n=110)** | **Nigeria (n=380)** | **Uganda (n=417)** | **Zambia (n=199)** | **Overall (n=1473)** |
| --- | --- | --- | --- | --- | --- | --- | --- | --- |
|  |  |  | **N (%)** | **N (%)** | **N (%)** | **N (%)** | **N (%)** | **N (%)** |
| **Demographics** | | | | | | | |  |
| **Age at baseline** | <40 | | 78 (21·3) | 10 (9·1) | 101 (26·6) | 115 (27·6) | 55 (27·6) | 359 (24·4) |
|  | 40-49 | | 98 (26·7) | 25 (22·7) | 112 (29·5) | 126 (30·2) | 52 (26·1) | 413 (28·0) |
|  | 50-59 | | 92 (25·1) | 31 (28·2) | 94 (24·7) | 93 (22·3) | 44 (22·1) | 354 (24·0) |
|  | 60+ | | 99 (27·0) | 44 (40·0) | 73 (19·2) | 83 (19·9) | 48 (24·1) | 347 (23·6) |
|  | Mean age (SD) | | 53 (15) | 57 (13) | 49 (12) | 48 (13) | 50 (15) | 50 (14) |
| **Education** | None/Primary school | | 191 (52·0) | 19 (17·3) | 100 (26·3) | 241 (57·8) | 103 (51·8) | 654 (44·4) |
|  | Secondary/high school | | 129 (35·1) | 49 (44·5) | 144 (37·9) | 126 (30·2) | 55 (27·6) | 503 (34·1) |
|  | Technical/University | | 47 (12·8) | 42 (38·2) | 136 (35·8) | 50 (12·0) | 41 (20·6) | 316 (21·5) |
| **Socioeconomic status** | Low | | 167 (45·5) | 0 (0·0) | 164 (43·2) | 245 (58·8) | 70 (35·2) | 646 (43·9) |
|  | Medium | | 127 (34·6) | 45 (40·9) | 162 (42·6) | 88 (21·1) | 71 (35·7) | 493 (33·5) |
|  | High | | 73 (19·9) | 65 (59·1) | 54 (14·2) | 84 (20·1) | 58 (29·1) | 334 (22·7) |
| **Residential area** | Urban | | 206 (56·1) | 100 (90·9) | 245 (64·5) | 110 (26·4) | 129 (64·8) | 790 (53·6) |
|  | Rural | | 161 (43·9) | 10 (9·1) | 135 (35·5) | 307 (73·6) | 70 (35·2) | 683 (46·4) |
| **Comorbidities** | | | | | | | |  |
| **HIV status** | Negative/Unknown status | | 315 (85·8) | 106 (96·4) | 371 (97·6) | 369 (88·5) | 167 (83·9) | 1328 (90·2) |
|  | Positive | | 52 (14·2) | 4 (3·6) | 9 (2·4) | 48 (11·5) | 32 (16·1) | 145 (9·8) |
| **BMI^*^** | <25 | | 122 (39·1) | 31 (30·4) | 142 (41·5) | 185 (47·0) | 72 (42·4) | 552 (41·8) |
|  | [25-30[ | | 93 (29·8) | 25 (24·5) | 107 (31·3) | 149 (37·8) | 53 (31·2) | 427 (32·3) |
|  | 30+ | | 97 (31·1) | 46 (45·1) | 93 (27·2) | 60 (15·2) | 45 (26·5) | 341 (25·8) |
|  | Mean BMI (SD) | | 27 (7) | 29 (7) | 27 (6) | 26 (5) | 27 (6) | 26 (6) |
| **Other comorbidities** | Yes | | 182 (49·6) | 71 (64·5) | 288 (75·8) | 130 (31·2) | 73 (36·7) | 744 (50·5) |
|  | No | | 185 (50·4) | 39 (35·5) | 92 (24·2) | 287 (68·8) | 126 (63·3) | 729 (49·5) |
| **Tobacco** | Never user | | 300 (81·7) | 65 (59·1) | 376 (98·9) | 399 (95·7) | 189 (95·0) | 1329 (90·2) |
|  | Ever user | | 67 (18·3) | 45 (40·9) | 4 (1·1) | 18 (4·3) | 10 (5·0) | 144 (9·8) |
| **Alcohol** | Never drank | | 183 (49·9) | 43 (39·1) | 173 (45·5) | 222 (53·2) | 127 (63·8) | 748 (50·8) |
|  | Ever drank | | 184 (50·1) | 67 (60·9) | 207 (54·5) | 195 (46·8) | 72 (36·2) | 725 (49·2) |
| **BC characteristics** | | | | | | | |  |
| **TNM stage^*^** | I | | 16 (4·4) | 22 (20·0) | 12 (3·4) | 32 (8·3) | 1 (0·6) | 83 (6·0) |
|  | II | | 112 (30·5) | 60 (54·5) | 76 (21·5) | 112 (29·2) | 73 (41·7) | 433 (31·2) |
|  | III | | 180 (49·0) | 21 (19·1) | 207 (58·6) | 176 (45·8) | 88 (50·3) | 672 (48·4) |
|  | IV | | 59 (16·1) | 7 (6·4) | 58 (16·4) | 64 (16·7) | 13 (7·4) | 201 (14·5) |
| **Treatment received^**^** | None | | 6 (2) | 1 (1) | 80 (28) | 24 (8) | 26 (18) | 137 (12) |
|  | Surgery | | 213 (72) | 90 (88) | 154 (54) | 181 (62) | 76 (52) | 714 (63) |
|  |  | if early-stage BC (TNM stage I/II) | 102 (82) | 74 (91) | 57 (66) | 94 (67) | 45 (65) | 372 (74) |
|  | Chemotherapy | | 236 (79) | 74 (73) | 141 (49) | 227 (77) | 107 (73) | 785 (70) |
|  | Surgery and chemotherapy (i.e., minimally adequate treatment) | | 179 (60) | 67 (66) | 101 (35) | 166 (57) | 63 (43) | 576 (51) |
|  | Endocrine therapy | | 224 (75) | 77 (76) | 108 (38) | 162 (55) | 61 (42) | 632 (56) |
|  | Radiotherapy | | 189 (63) | 66 (65) | 9 (3) | 51 (17) | 35 (24) | 350 (31) |

BMI = Body mass index; BC = Breast cancer; SD = Standard deviation.

*****BMI and TNM stage information were missing for 153 and 84 women, respectively.

**This analysis was restricted to women with non-metastatic disease (i.e., TNM I/II/III) with a known treatment status (n=1129).

# **Appendix 2. Supplementary data for the main analysis**

| **ABC-DO country and race** | **Namibia** | | | **Nigeria** | **Uganda** | **Zambia** | **Overall** |
| --- | --- | --- | --- | --- | --- | --- | --- |
|  | Non-Black, n=110 | Black, n=367 | All, n=477 | n=380 | n=417 | n=199 | n=1473 |
|  | **N (%)** | **N (%)** | **N (%)** | **N (%)** | **N (%)** | **N (%)** | **N (%)** |
| **GBCI pillar 1, among known stage at diagnosis** | **n=110** | **n=367** | **n=477** | **n=353** | **n=384** | **n=175** | **n=1389** |
| **Staging methods** |  |  |  |  |  |  |  |
| Imaging only | 38 (34·5) | 27 (7·4) | 65 (13·6) | 94 (26·6) | 206 (53·6) | 93 (53·1) | 458 (33·0) |
| Clinical only | 1 (0·9) | 10 (2·7) | 11 (2·3) | 177 (50·1) | 29 (7·6) | 16 (9·1) | 233 (16·8) |
| Surgery only | 3 (2·7) | 6 (1·6) | 9 (1·9) | 0 (0) | 1 (0·3) | 0 (0) | 10 (0·7) |
| Imaging and Clinical | 29 (26·4) | 171 (46·6) | 200 (41·9) | 10 (2·8) | 48 (12·5) | 15 (8·6) | 273 (19·7) |
| Imaging and Surgery | 24 (21·8) | 65 (17·7) | 89 (18·7) | 1 (0·3) | 6 (1·6) | 8 (4·6) | 104 (7·5) |
| Clinical and surgery | 1 (0·9) | 5 (1·4) | 6 (1·3) | 5 (1·4) | 3 (0·8) | 0 (0) | 14 (1·0) |
| Clinical, Imaging and Surgery | 6 (5·5) | 14 (3·8) | 20 (4·2) | 0 (0) | 0 (0) | 0 (0) | 20 (1·4) |
| Unknown staging method | 8 (7·3) | 69 (18·8) | 77 (16·1) | 66 (18·7) | 91 (23·7) | 43 (24·6) | 277 (19·9) |
| **GBCI pillar 2, among histologically diagnosed** | **n=110** | **n=367** | **n=477** | **n=205** | **n=360** | **n=180** | **n=1222** |
| Date of first presentation to the healthcare system prior to the date of diagnosis | 104 (94·5) | 351 (95·6) | 455 (95·4) | 149 (72·7) | 342 (95·0) | 162 (90·0) | 1108 (90·7) |
| **Type of diagnosis date*** |  |  |  |  |  |  |  |
| Date of pathology report | 104 (100) | 349 (99·4) | 453 (99·6) | 136 (91·3) | 332 (97·1) | 154 (95·1) | 1075 (97·0) |
| Date of laboratory receipt | 0 (0) | 1 (0·3) | 1 (0·2) | 10 (6·7) | 1 (0·3) | 6 (3·7) | 18 (1·6) |
| Date of biopsy sampling | 0 (0) | 1 (0·3) | 1 (0·2) | 2 (1·3) | 2 (0·6) | 0 (0) | 5 (0·45) |
| Histology (no info on type of date) | 0 (0) | 0 (0) | 0 (0) | 1 (0·7) | 7 (2·0) | 2 (1·2) | 10 (0·90) |
| **Type of first provider*** |  |  |  |  |  |  |  |
| Formal sector | 104 (100) | 350 (99·7) | 454 (99·8) | 141 (94·6) | 313 (91·5) | 162 (100) | 1070 (96·6) |
| Primary care | 85 (81·7) | 189 (53·8) | 274 (60·2) | 8 (5·4) | 102 (29·8) | 62 (38·3) | 446 (40·3) |
| Secondary/tertiary | 19 (18·3) | 161 (45·9) | 180 (39·6) | 133 (89·3) | 211 (61·7) | 100 (61·7) | 624 (56·3) |
| Informal sector | 0 (0) | 1 (0·3) | 1 (0·2) | 8 (5·4) | 29 (8·5) | 0 (0) | 38 (3·4) |
| **First presentation to histological diagnosis, median (IQR), in days*** | 32 (16, 96) | 76 (26, 230) | 63 (23, 197) | 63 (26, 241) | 187 (68, 455) | 100 (48, 253) | 100 (32, 295) |
| First presentation to the healthcare system to biopsy sampling, median (IQR), in days | 21 (8, 84) | 64 (16, 217) | 49 (12, 187) | 53 (11, 211) | 148 (71, 454) | 69 (29, 239) | 63 (15, 225) |
| Biopsy sampling to laboratory receipt, median (IQR), in days | 0 (0, 1) | 0 (0, 1) | 0 (0, 1) | 0 (0, 0) | 0 (0, 1) | 0 (0, 2) | 0 (0, 1) |
| Laboratory receipt to pathology report, median (IQR), in days | 6 (2, 11) | 7 (4, 12) | 7 (3, 12) | 12 (6, 25) | 4 (2, 7) | 13 (7, 32) | 7 (3, 13) |
| **GBCI pillar 3** |  |  |  |  |  |  |  |
| **Among women with non-metastatic disease, no. women with known treatment status** | **n=102** | **n=298** | **n=400** | **n=288** | **n=294** | **n=147** | **n=1129** |
| Died within 6 months of baseline | 1 (1·0) | 11 (3·7) | 12 (3·0) | 32 (11·1) | 14 (4·8) | 13 (8·8) | 71 (6·3) |
| Known ER status | 101 (99·0) | 291 (97·7) | 392 (98·0) | 36 (12·5) | 38 (12·9) | 42 (28·6) | 508 (45·0) |
| Any treatment initiated | 101 (99·0) | 292 (98·0) | 393 (98·3) | 208 (72·2) | 270 (91·8) | 121 (82·3) | 992 (87·9) |
| Known treatment initiation date | 101 (100) | 292 (100) | 393 (100) | 208 (100) | 270 (100) | 121 (100) | 992 (100) |
| **First contact with the healthcare system prior to treatment initiation** | **101 (99·0)** | **296 (99·3)** | **397 (99·3)** | **272 (94·4)** | **288 (98·0)** | **145 (98·6)** | **1102 (97·6)** |
| Treatment initiated within 90 days of first presentation to the healthcare system | 67 (66·3) | 125 (42·2) | 192 (48·4) | 71 (26·1) | 39 (13·5) | 34 (23·4) | 336 (30·5) |
| **Among women with metastatic disease** | **n=16** | **n=101** | **n=117** | **n=64** | **n=70** | **n=15** | **n=266** |
| No palliative care information | 2 (12·5) | 30 (29·7) | 32 (27·4) | 46 (71·9) | 54 (77·1) | 11 (73·3) | 143 (53·8) |
| Known palliative systemic therapy status only | 9 (56·3) | 29 (28·7) | 38 (32·5) | 1 (1·6) | 1 (1·4) | 1 (6·7) | 41 (15·4) |
| Known palliative supportive care status only | 3 (18·8) | 19 (18·8) | 22 (18·8) | 11 (17·2) | 13 (18·6) | 2 (13·3) | 48 (18·0) |
| Both palliative systemic therapy and supportive care statuses known | 2 (12·5) | 23 (22·8) | 25 (21·4) | 6 (9·4) | 2 (2·9) | 1 (6·7) | 34 (12·8) |
| **Palliative treatment received** |  |  |  |  |  |  |  |
| Any | 14 (87·5) | 71 (70·3) | 85 (72·6) | 18 (28·1) | 13 (18·6) | 4 (26·7) | 120 (45·1) |
| Palliative systemic therapy | 11 (68·8) | 52 (51·5) | 63 (53·8) | 7 (10·9) | 3 (4·3) | 2 (13·3) | 75 (28·2) |
| Palliative symptomatic treatment | 5 (31·3) | 42 (41·6) | 47 (40·2) | 17 (26·6) | 12 (17·1) | 3 (20·0) | 79 (29·7) |
| Palliative psychological support | 5 (31·3) | 37 (36·6) | 42 (35·9) | 12 (18·8) | 7 (10·0) | 3 (20·0) | 64 (24·1) |
| Palliative social support | 3 (18·8) | 14 (13·9) | 17 (14·5) | 2 (3·1) | 2 (2·9) | 1 (6·7) | 22 (8·3) |

ABC-DO: African Breast Cancer – Disparities in Outcomes; GBCI: Global breast cancer initiative; IQR: interquartile range.

*Among women with a date of first presentation to the healthcare system prior to the date of diagnosis

# **Appendix 3. Supplementary data for the sensitivity analyses**

| **ABC-DO country** | **Namibia** | | | **Nigeria** | | **Uganda** | | **Zambia** | | **Overall** | |  |
| --- | --- | --- | --- | --- | --- | --- | --- | --- | --- | --- | --- | --- |
|  | Non-Black, n=110 | Black, n=367 | All, n=477 | | n=380 | | n=417 | | n=199 | | n=1473 | |
|  | **N (%)** | **N (%)** | **N (%)** | | **N (%)** | | **N (%)** | | **N (%)** | | **N (%)** | |
| **GBCI pillar 2, no. women histologically diagnosed** | **n=110** | **n=367** | **n=477** | | **n=205** | | **n=360** | | **n=180** | | **n=1222** | |
| **Type of diagnosis date** |  |  |  | |  | |  | |  | |  | |
| Date of pathology report | 110 (100) | 365 (99·5) | 475 (99·6) | | 190 (92·7) | | 349 (96·9) | | 171 (95·0) | | 1185 (97·0) | |
| Date of laboratory receipt | 0 (0) | 1 (0·3) | 1 (0·2) | | 11 (5·4) | | 1 (0·3) | | 7 (3·9) | | 20 (1·6) | |
| Date of biopsy sampling | 0 (0) | 1 (0·3) | 1 (0·2) | | 3 (1·5) | | 3 (0·8) | | 0 (0) | | 7 (0·6) | |
| Biopsy (no info on type of date) | 0 (0) | 0 (0) | 0 (0) | | 1 (0·5) | | 7 (1·9) | | 2 (1·1) | | 10 (0·8) | |
| **Type of first provider** |  |  |  | |  | |  | |  | |  | |
| Formal sector | 110 (100) | 366 (99·7) | 476 (99·8) | | 197 (96·1) | | 331 (91·9) | | 179 (99·4) | | 1183 (96·8) | |
| Primary care | 89 (80·9) | 194 (52·9) | 283 (59·3) | | 8 (3·9) | | 105 (29·2) | | 64 (35·6) | | 460 (37·6) | |
| Secondary/tertiary | 21 (19·1) | 172 (46·9) | 193 (40·5) | | 189 (92·2) | | 226 (62·8) | | 115 (63·9) | | 723 (59·2) | |
| Informal sector | 0 (0) | 1 (0·3) | 1 (0·2) | | 8 (3·9) | | 29 (8·1) | | 1 (0·6) | | 39 (3·2) | |
| **First presentation to histological diagnosis, median (IQR), in days** | 31 (14, 85) | 73 (24, 224) | 58 (22, 187) | | 38 (13, 164) | | 176 (57, 424) | | 88 (41, 238) | | 81 (26, 266) | |
| First presentation to the healthcare system to biopsy sampling, median (IQR), in days | 20 (6, 69) | 59 (13, 206) | 40 (9, 175) | | 14 (0, 119) | | 143 (59, 454) | | 50 (9, 201) | | 47 (8, 189) | |
| Biopsy sampling to laboratory receipt, median (IQR), in days | 0 (0, 1) | 0 (0, 1) | 0 (0, 1) | | 0 (0, 0) | | 0 (0, 1) | | 0 (0, 2) | | 0 (0, 1) | |
| Laboratory receipt to pathology report, median (IQR), in days | 6 (2, 10) | 7 (4, 13) | 7 (3, 12) | | 11 (6, 25) | | 4 (2, 7) | | 13 (7, 32) | | 7 (3, 13) | |
| **GBCI pillar 3** |  |  |  | |  | |  | |  | |  | |
| **Among women with non-metastatic disease, no. with known treatment status** | **n=101** | **n=287** | **n=388** | | **n=256** | | **n=280** | | **n=134** | | **n=1058** | |
| Known ER status | 100 (99·0) | 280 (97·6) | 380 (97·9) | | 35 (13·7) | | 37 (13·2) | | 40 (29·9) | | 492 (46·5) | |
| Any treatment initiated | 101 (100) | 283 (98·6) | 384 (99·0) | | 196 (76·6) | | 262 (93·6) | | 117 (87·3) | | 959 (90·6) | |
| **First contact with the healthcare system prior to treatment initiation** | **100 (99·0)** | **285 (99·3)** | **385 (99·2)** | | **243 (94·9)** | | **274 (97·9)** | | **132 (98·5)** | | **935 (88·4)** | |
| Treatment initiated within 90 days of first presentation to the healthcare system | 67 (67·0) | 120 (42·1) | 187 (48·6) | | 66 (27·2) | | 37 (13·5) | | 33 (25·0) | | 323 (34·5) | |
| **Among women with metastatic disease** | **n=17** | **n=115** | **n=132** | | **n=101** | | **n=94** | | **n=40** | | **n=367** | |
| No palliative care information | 3 (17·6) | 36 (31·3) | 39 (29·5) | | 75 (74·3) | | 72 (76·6) | | 34 (85·0) | | 220 (59·9) | |
| Known palliative systemic therapy status only | 9 (52·9) | 34 (29·6) | 43 (32·6) | | 1 (1·0) | | 2 (2·1) | | 2 (5·0) | | 48 (13·1) | |
| Known palliative supportive care status only | 3 (17·6) | 19 (16·5) | 22 (16·7) | | 19 (18·8) | | 18 (19·1) | | 3 (7·5) | | 62 (16·9) | |
| Both palliative systemic therapy and supportive care statuses known | 2 (11·8) | 26 (22·6) | 28 (21·2) | | 6 (5·9) | | 2 (2·1) | | 1 (2·5) | | 37 (10·1) | |
| **Palliative treatment received** |  |  |  | |  | |  | |  | |  | |
| Any | 14 (82·4) | 79 (68·7) | 93 (70·5) | | 26 (25·7) | | 16 (17·0) | | 6 (15·0) | | 141 (38·4) | |
| Palliative systemic therapy | 11 (64·7) | 60 (52·2) | 71 (53·8) | | 7 (6·9) | | 4 (4·3) | | 3 (7·5) | | 85 (23·2) | |
| Palliative symptomatic treatment | 5 (29·4) | 45 (39·1) | 50 (37·9) | | 25 (24·8) | | 14 (14·9) | | 4 (10·0) | | 93 (25·3) | |
| Palliative psychological support | 5 (29·4) | 39 (33·9) | 44 (33·3) | | 15 (14·9) | | 8 (8·5) | | 3 (7·5) | | 70 (19·1) | |
| Palliative social support | 3 (17·6) | 14 (12·2) | 17 (12·9) | | 2 (2·0) | | 2 (2·1) | | 1 (2·5) | | 22 (6·0) | |

ABC-DO: African Breast Cancer – Disparities in Outcomes; GBCI: Global breast cancer initiative; IQR: interquartile range.

# **Appendix 4. GBCI pillar key performance indicators estimates, by country and race, in ABC-DO (sensitivity analysis)**

ABC-DO: African Breast Cancer – Disparities in Outcomes; GBCI: Global breast cancer initiative; KPI: Key Performance Indicator.

# **Appendix 5. Patterns of co-KPI achievement, in ABC-DO**

| **Patterns of co-KPI achievement** | **KPI-1** | **KPI-2** | **KPI-3** | **No. KPIs achieved** | **Stage I, II, III** | **Stage IV*** |
| --- | --- | --- | --- | --- | --- | --- |
|  |  |  |  |  | **N (col %)** | **N (col %)** |
| No KPI achieved | N | N | N | 0 | 284 (24) | 94 (47) |
| KPI-2 only | N | Y | N | 1 | 139 (12) | 58 (29) |
| KPI-1 +- KPI-2 - KPI-3 | Y | N | N | 1 | 191 (16) | 0 (0) |
|  | Y | Y | N | 2 | 134 (11) | 0 (0) |
| KPI-3 +- KPI-2 - KPI-1 | N | N | Y | 1 | 37 (3·1) | N/A |
|  | N | Y | Y | 2 | 26 (2·2) | N/A |
| KPI-1 +- KPI-2 + KPI-3 | Y | N | Y | 2 | 29 (2·4) | N/A |
|  | Y | Y | Y | 3 | 39 (3·3) | N/A |
| At least one KPI not calculated |  |  |  | NK | 309 (26) | 49 (24) |
| **Total** | | | | | **1188 (100)** | **201 (100)** |

* For women affected with metastatic disease (i.e., breast cancer stage IV), the analysis restricted to KPI-1 and KPI-2.
